# Supplementary material for: Effects of arsenic on the topology and solubility of promyelocytic leukemia (PML)-nuclear bodies
Source: PLoS One. 2022 May 20;17(5):e0268835. doi: 10.1371/journal.pone.0268835 (PMC9122205; doi:10.1371/journal.pone.0268835)
Supplement: S1 Fig — Live GFP images were captured during cell division by confocal laser scanning microscopy in z-stacking mode. The upper and middle 8 panels show GFP alone and the corresponding GFP-bright field overlaid images, respectively. The lower 8 panels show enlarged and intensified GFP images of a peri-nuclear PML aggregate indicated with arrows on the top four panels with four additional time-lapsed GFP images. The time counter is shown in the right upper corner of each panel. The peri-nuclear PML aggregates appear to be comprised of 2–4 toroids. The nascent small PML-NBs (arrowheads) appeared as the daughter cells spread. (PDF) [file pone.0268835.s001.pdf]

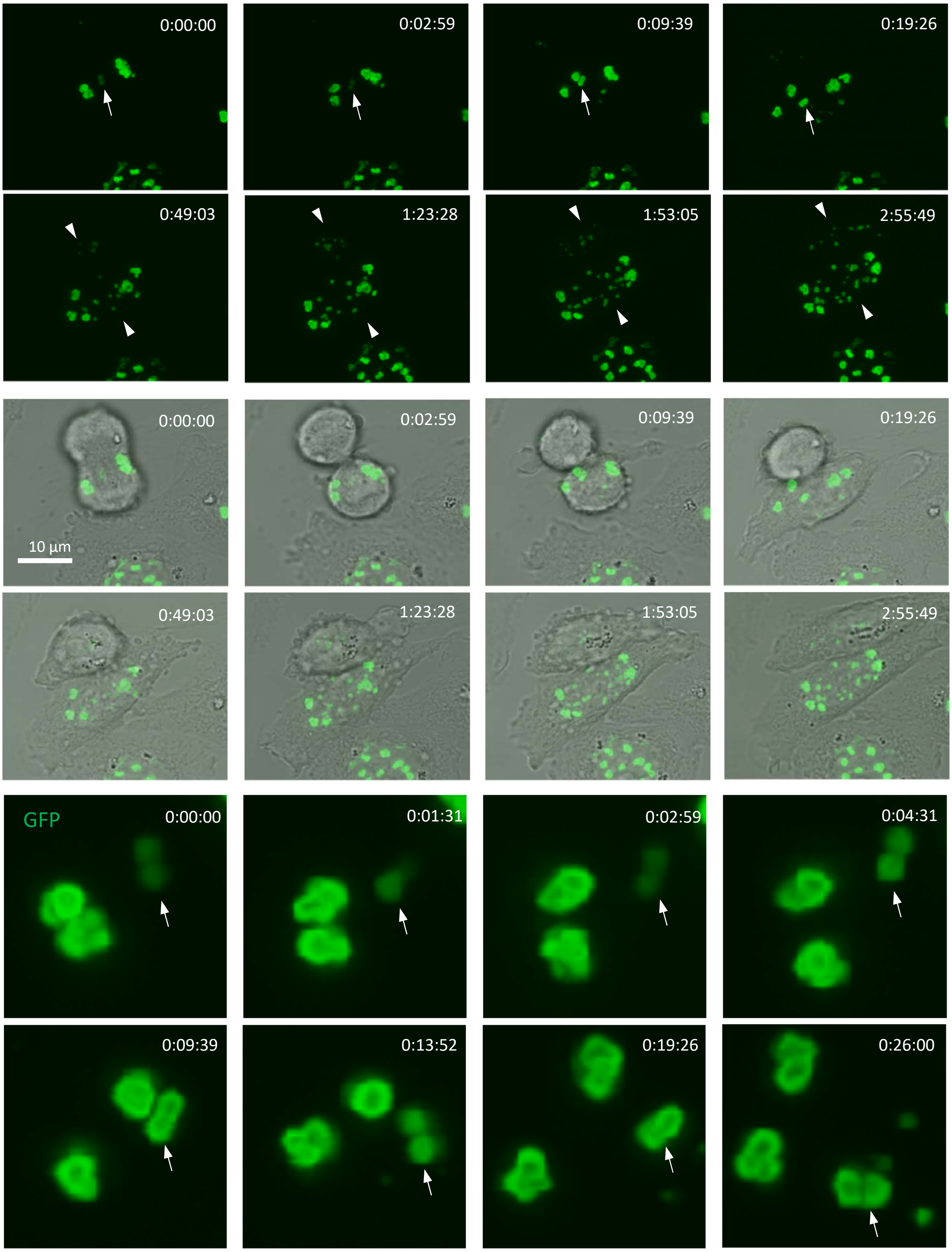

S1 Fig., Emergence and uneven partitioning of peri-nuclear PML aggregates in dividing untreated CHOGFPPML cells. Live GFP images were captured during cell division by confocal laser scanning microscopy in z-stacking mode. The upper and middle 8 panels show GFP alone and the corresponding GFP-bright field overlaid images, respectively. The lower 8 panels show enlarged and intensified GFP images of a peri-nuclear PML aggregate indicated with arrows on the top four panels with four additional time-lapsed GFP images. The time counter is shown in the right upper corner of each panel. The peri-nuclear PML aggregates appear to be comprised of 2-4 toroids. The nascent small PML-NBs (arrowheads) appeared as the daughter cells spread.
