# Supplementary material for: Effects of arsenic on the topology and solubility of promyelocytic leukemia (PML)-nuclear bodies
Source: PLoS One. 2022 May 20;17(5):e0268835. doi: 10.1371/journal.pone.0268835 (PMC9122205; doi:10.1371/journal.pone.0268835)
Supplement: S2 Fig — Image capture started 30 min after the addition of As3+. The upper and lower 10 panels show GFP images alone and the corresponding GFP-bright field overlaid images, respectively. The nascent small PML-NBs (arrowheads) appeared as the daughter cells spread. See also the legend to S1 Fig. (PDF) [file pone.0268835.s002.pdf]

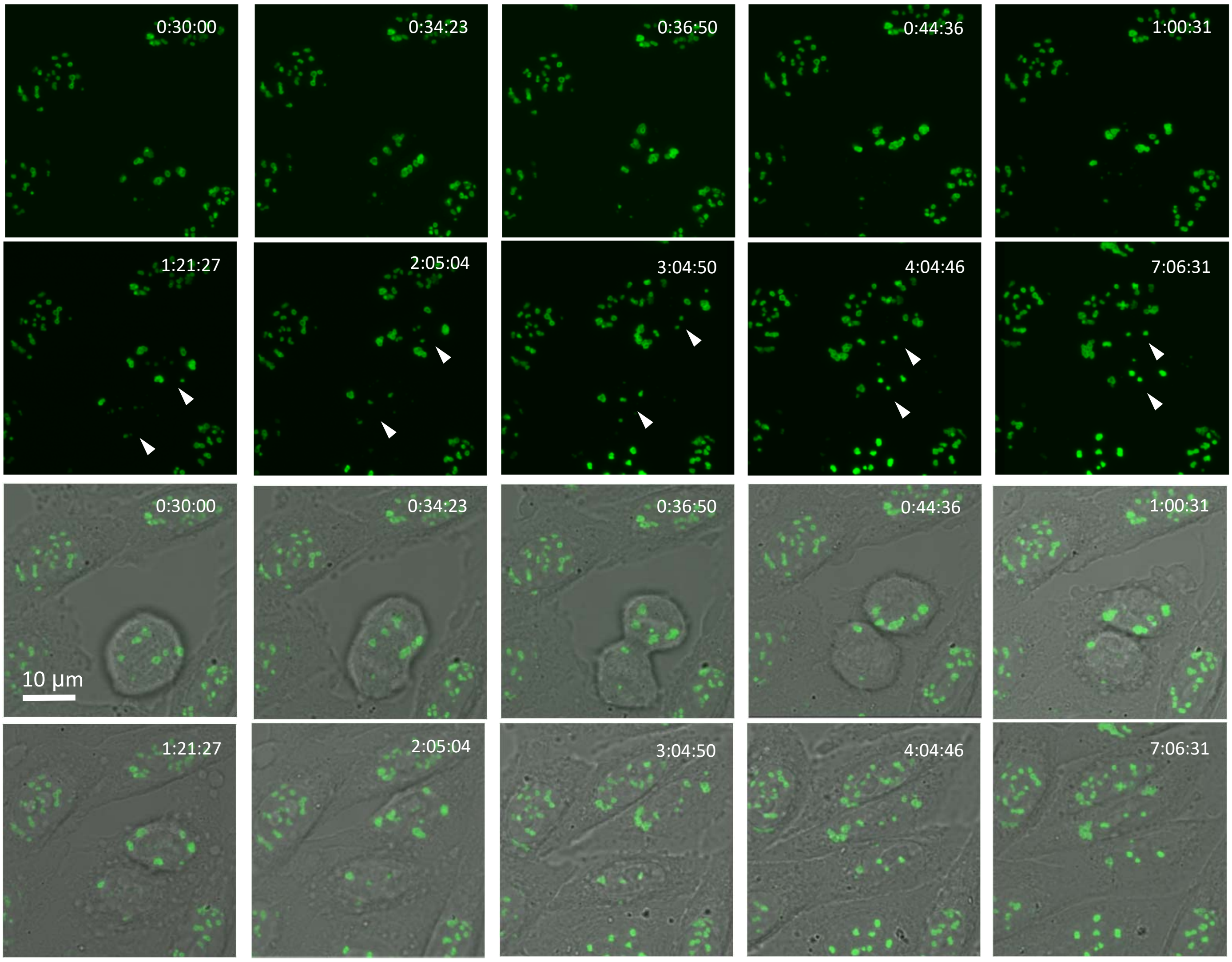

S2 Fig., Uneven partitioning of peri-nuclear PML aggregates in As^{3+}-exposed CHOGFPPML cells. Image capture started 30 min after the addition of As^{3+}. The upper and lower 10 panels show GFP images alone and the corresponding GFP-bright field overlaid images, respectively. The nascent small PML-NBs (arrowheads) appeared as the daughter cells spread. See also the legend to **S1 Fig.**.
