# Supplementary material for: Effects of arsenic on the topology and solubility of promyelocytic leukemia (PML)-nuclear bodies
Source: PLoS One. 2022 May 20;17(5):e0268835. doi: 10.1371/journal.pone.0268835 (PMC9122205; doi:10.1371/journal.pone.0268835)
Supplement: S3 Fig — The proteasomal inhibitory effect was assayed using a 20S proteasome assay kit. Briefly, the fluorescence (Ex/Em, 360/460 nm) of the reaction mixture (proteasome, Suc-LLVY-AMC, and SDS) was monitored every 1 min in the presence or absence of As3+, Sb3+, Bi3+, Cd2+, or epoxomicin (a positive control) at 30°C for 20 min using a microplate reader (Infinite M Plex, TECAN, Männedorf, Switzerland). As3+, Sb3+, Bi3+, and Cd2+ were added to the reaction mixture at a final concentration of 30 μM. The fluorescence was increased linearly and the increment of fluorescence in 20 min was used for evaluation of relative inhibitory activity of proteasome. Data are presented as means ± SEM of 4 replicate measurements. (PDF) [file pone.0268835.s003.pdf]

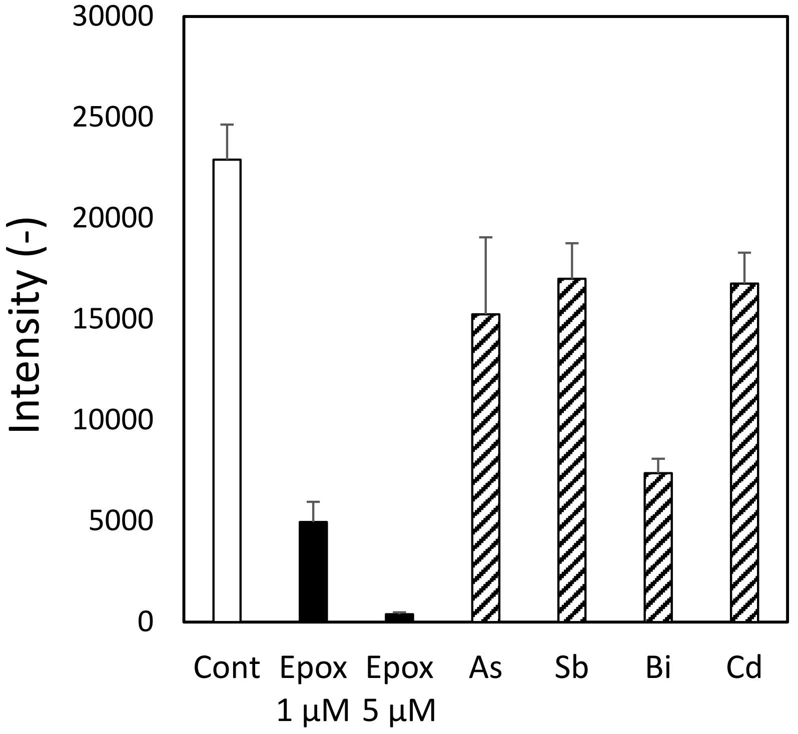


**S3 Fig., Inhibition of proteasomal activity by $As^{3+}$, $Sb^{3+}$, $Bi^{3+}$, and $Cd^{2+}$.** The proteasomal inhibitory effect was assayed using a 20S proteasome assay kit. Briefly, the fluorescence (Ex/Em, 360/460 nm) of the reaction mixture (proteasome, Suc-LLVY-AMC, and SDS) was monitored every 1 min in the presence or absence of $As^{3+}$, $Sb^{3+}$, $Bi^{3+}$, $Cd^{2+}$, or epoxomicin (a positive control) at 30°C for 20 min using a microplate reader (Infinite M Plex, TECAN, Männedorf, Switzerland). $As^{3+}$, $Sb^{3+}$, $Bi^{3+}$, and $Cd^{2+}$ were added to the reaction mixture at a final concentration of 30 µM. The fluorescence was increased linearly and the increment of fluorescence in 20 min was used for evaluation of relative inhibitory activity of proteasome. Data are presented as means ± SEM of 4 replicate measurements.
