# Supplementary material for: Effects of arsenic on the topology and solubility of promyelocytic leukemia (PML)-nuclear bodies
Source: PLoS One. 2022 May 20;17(5):e0268835. doi: 10.1371/journal.pone.0268835 (PMC9122205; doi:10.1371/journal.pone.0268835)
Supplement: S4 Fig — The cells were exposed to 3 μM As3+ for 24 h or left untreated. The As3+-exposed cells were lysed with the RIPA buffer immediately or washed and further cultured in As3+-free culture medium further for 8 or 24 h. 1, untreated; 2, 24 h exposure to As3+; 3, 24 h exposure to As3+and 8 h recovery in As3+-free culture medium; 4, 24 h exposure to As3+and 24 h recovery in As3+-free culture medium. An open arrowhead indicates SUMO2/3 monomers. The unconjugated GFPPML and GFPPML conjugated with SUMO2/3 in the RIPA-insoluble fraction (Ins) decreased during the culture in As3+-free culture medium. (PDF) [file pone.0268835.s004.pdf]

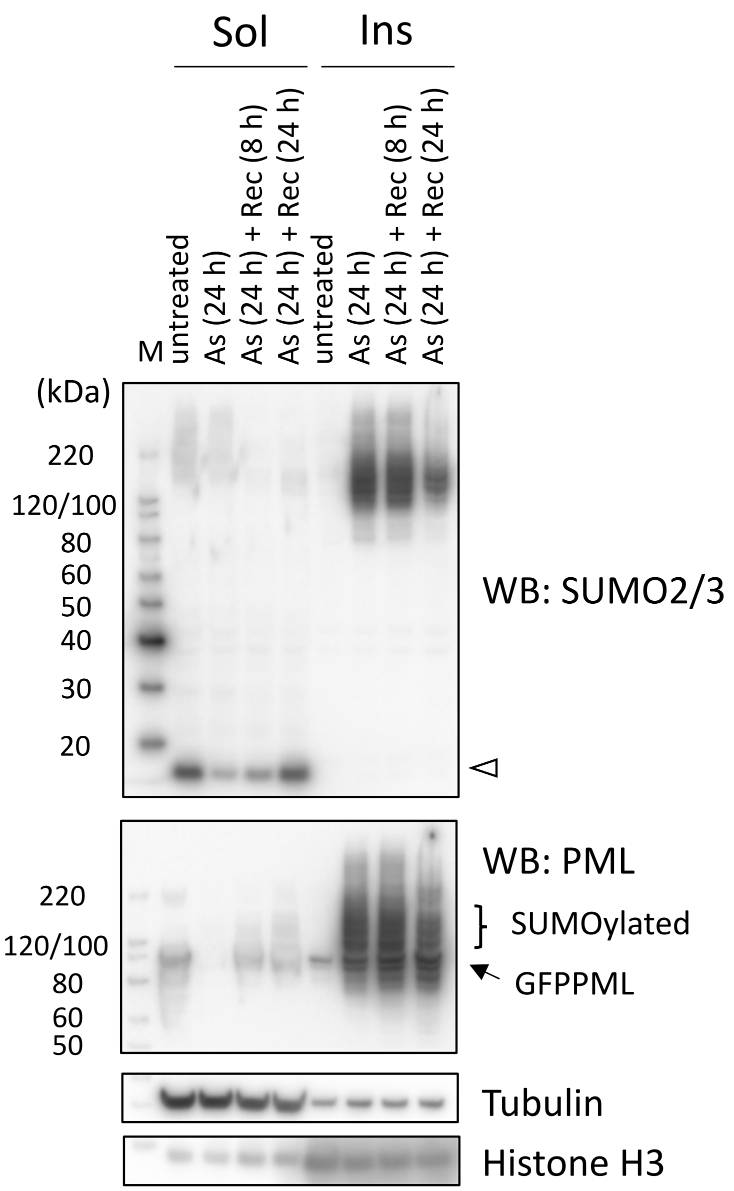

S4 Fig., Recovery of SUMO2/3 and GFPPML proteins in the RIPA-soluble fraction (Sol) from 24 h-exposure to As^{3+} in HEKGFPPML cells. The cells were exposed to 3 μM As^{3+} for 24 h or left untreated. The As^{3+}-exposed cells were lysed with the RIPA buffer immediately or washed and further cultured in As^{3+}-free culture medium further for 8 or 24 h. 1, untreated; 2, 24 h exposure to As^{3+}; 3, 24 h exposure to $As^{3+}$and 8 h recovery in As^{3+}-free culture medium; 4, 24 h exposure to $As^{3+}$and 24 h recovery in As^{3+}-free culture medium. An open arrowhead indicates SUMO2/3 monomers. The unconjugated GFPPML and GFPPML conjugated with SUMO2/3 in the RIPA-insoluble fraction (Ins) decreased during the culture in As^{3+}-free culture medium.
