# Supplementary material for: Effects of arsenic on the topology and solubility of promyelocytic leukemia (PML)-nuclear bodies
Source: PLoS One. 2022 May 20;17(5):e0268835. doi: 10.1371/journal.pone.0268835 (PMC9122205; doi:10.1371/journal.pone.0268835)
Supplement: S5 Fig — Time-course changes in the number of viable ML792-exposed cells (A) and the inhibitory effects of ML792 on the cytotoxicity of TAK243 (B) in HEKGFPPML cells. (A) The cells were exposed to 0.1% DMSO (open columns) or 20 μM ML792 (hatched columns) for 2, 4, 8, 15 h. The number of viable cells was assayed using WST-8 agent. Data are presented as means ± SEM of five wells. (B) The cells were exposed to 0, 2.5, 5, and 10 μM TAK243 for 24 h in the presence or absence of 20 μM ML792. Data were analyzed by two-way ANOVA followed by Tukey’s multiple comparison. The viability of ML792-treated cells was significantly elevated compared to 0.1% DMSO-treated cells in the presence of 10 μM TAK243. (PDF) [file pone.0268835.s005.pdf]

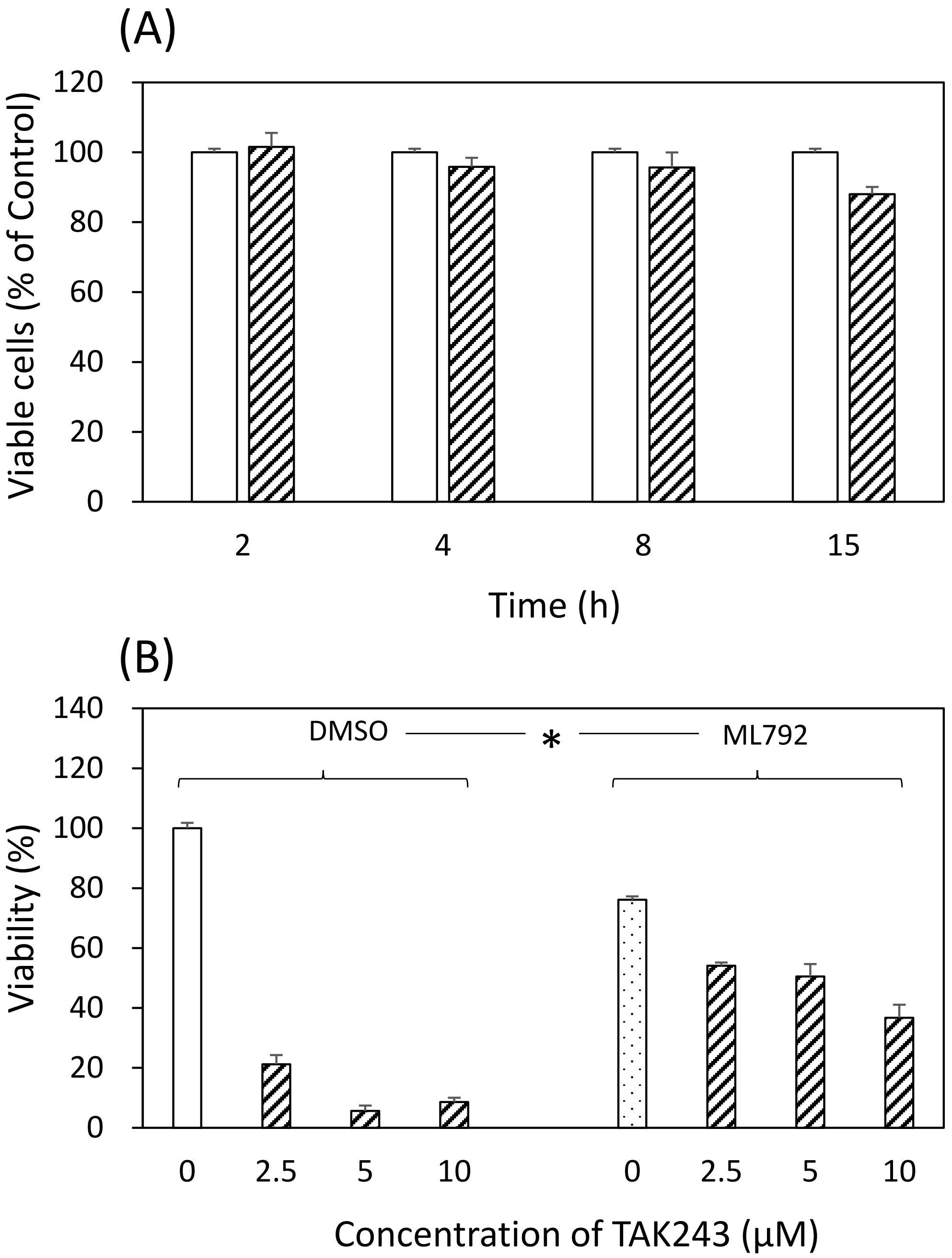


**S5 Fig., Time-course changes in the number of viable ML792-exposed cells (A) and the inhibitory effects of ML792 on the cytotoxicity of TAK243 (B) in HEKGFPPML cells**. (A) The cells were exposed to 0.1% DMSO (open columns) or 20 μM ML792 (hatched columns) for 2, 4, 8, 15 h. The number of viable cells was assayed using WST-8 agent. Data are presented as means ± SEM of five wells. (B) The cells were exposed to 0, 2.5, 5, and 10 μM TAK243 for 24 h in the presence or absence of 20 μM ML792. Data were analyzed by two-way ANOVA followed by Tukey's multiple comparison. The viability of ML792-treated cells was significantly elevated compared to 0.1% DMSO-treated cells in the presence of 10 μM TAK243.
