# Supplementary figures and images for: Effects of arsenic on the topology and solubility of promyelocytic leukemia (PML)-nuclear bodies
Source: PLoS One. 2022 May 20;17(5):e0268835. doi: 10.1371/journal.pone.0268835 (PMC9122205; doi:10.1371/journal.pone.0268835)

Fig. 5 (SUMO2/3)

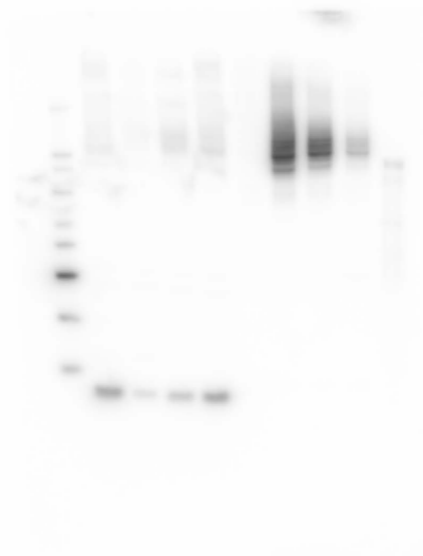

Fig. 5

Fig.

Fig. 6A (SUMO2/3)

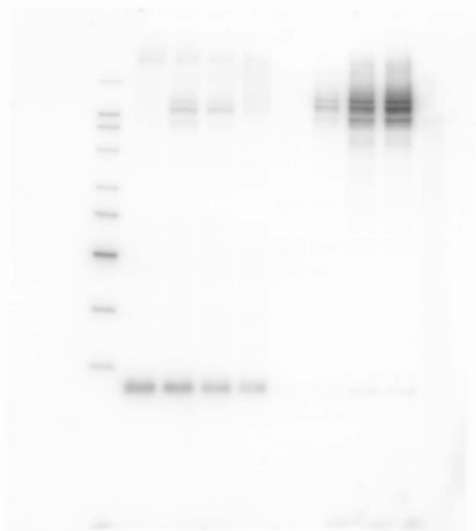

Supplement: S1 Raw images — (PDF) [file pone.0268835.s006.pdf]
