# Supplementary material for: Effects of arsenic on the topology and solubility of promyelocytic leukemia (PML)-nuclear bodies
Source: PLoS One. 2022 May 20;17(5):e0268835. doi: 10.1371/journal.pone.0268835 (PMC9122205; doi:10.1371/journal.pone.0268835)
Supplement: Attachment — Submitted filename: Response to Reviewers.pdf [file pone.0268835.s009.pdf]

Responses to Journal Requirements

Q1. Please review your reference list to ensure that it is complete and correct. If you have cited papers that have been retracted, please include the rationale for doing so in the manuscript text, or remove these references and replace them with relevant current references. Any changes to the reference list should be mentioned in the rebuttal letter that accompanies your revised manuscript. If you need to cite a retracted article, indicate the article's retracted status in the References list and also include a citation and full reference for the retraction notice.

A1. Our recently published paper has been added to the reference list of the revised manuscript.

35. Hirano S, Udagawa O. SUMOylation regulates the number and size of promyelocytic leukemia-nuclear bodies (PML-NBs) and arsenic perturbs SUMO dynamics on PML by insolubilizing PML in THP-1 cells. Arch Toxicol. 2022;96(2):545-58. doi: 10.1007/s00204-021-03195-w. PubMed PMID: 35001170.

We are sure that the reference list meets PLOS ONE's style requirements.

.

Point-by-point details as to how the manuscript has been revised

Reviewer #1:

Q0: The authors have addressed the concerns made by the 2 reviewers, but unfortunately in the minimal way: the changes mostly concern the text of the manuscript, and fail to really clarify the biological significance of the presented findings and to make the manuscript easier to read. Overall, I still find the manuscript weak, both in the solidity and in the originality of the conclusions that can be drawn from the experiments.

A0: We believe that our present manuscript contains several new interesting findings. We have stressed on the following points in our 2nd revised manuscript.

(1) PML-NBs were toroidal in CHO cells and granular dot-like in HEK cells. The shapes of PML bodies were not affected even after nuclear envelope breakdown.

(2) SUMOylation of PML-VI is not required for the solubility shift of this protein in As^{3+}-exposed cells.

(3) In contrast to the remarkable biochemical changes, the size and number of PML-NBs were not significantly changed by 2-h exposure to As^{3+}. However, PML-NBs were agglomerated after prolonged exposure to As^{3+}.

(4) Inhibition of SUMOylation rather than As^{3+}-induced robust SUMOylation of PML regulated the number and size of PML-NBs.

We have rewritten CONCLUSION extensively. Now it reads as follows.

"In summary, topological features of PML-NBs were different between CHO and HEK cells, although biochemical responses of PML to As^{3+} such as solubility shift and SUMOylation were almost the same between these two different cells. The solubility shift of PML occurred prior to SUMOylation. The number, size, and shape of PML-NBs were not changed by 2-h exposure to As^{3+}. However, the prolonged exposure to As^{3+} or Sb^{3+} caused agglomeration of PML-NBs which is reversed after removal of these metalloids from the culture medium. The present results warrant the importance of SUMO-dependent and -independent dynamic nature of PML-NBs which are implicated in phase separation cell biology and human diseases."

Q1: first paragraph: the residence-time of PML ranges --> the residence-time of PML in PML bodies ranges...

A1: We have changed the corresponding sentence as follows: "The residence-time of PML in PML nuclear bodies (PML-NBs) ranges from several minutes to one hour, whereas that of other PML-NB client proteins such as Sp100 and Daxx varies from several seconds to one minute [6], which led to the notion that PML is a scaffold protein for PML-NBs."

Q2: beginning of second paragraph: define B1 and B2 domains

A2: We have replaced "both B1 and B2 domains" with "both B-box zinc finger domains (B_1 and B_2)".

Q3: second paragraph: The unique subdomain appears to play a role in PML tetramer --> tetramerization? Same sentence: the following subsequent PML-NB formation --> remove either following or subsequent as both terms are redundant

A3: We have replaced tetramer with tetramerization and removed "following" from the sentence.

Q4: Material and methods: the real term is confoncal laser scanning microscopy and not confoncal laser microscopy

A4: We have added "scanning" after "confocal laser" in the text including figure legends.

Q5: Results: the definition of what the authors consider toroidal or irregular-shaped punctates is still unclear: this makes it difficult to visualize in the images the structures

referred to in the text. Typically, I don't understand how the authors can conclude that in HEKGFPPML cells, the "occasionally found" toroidal PML-NB are those that dissipate in small PML-NBs.

A5: We agree that the term “irregular-shaped” is ambiguous and hard to picture the shape. We have decreased terms relating to PML-NBs. Only two words are now used to depict the different shape of PML bodies in the revised manuscript, toroidal PML-NBs for CHO cells and granular dot-like PML-NBs for HEK cells. A term “PML aggregates” is used for peri-nuclear PML bodies which some other scientists call MAPPs or CyPNs. We wish “PML aggregates” could be replaced with PML-NBs. However, “PML aggregates” are not located in the nucleus and cannot be called nuclear bodies. Thus, “peri-nuclear PML aggregates” is used in the revised manuscript. We also use another term “PML agglomerates” which were observed in the nuclei of cells after prolonged exposure to As^{3+} or Sb^{3+}. The agglomeration of PML-NBs was a reversible process, and the clumped PML-NBs were separated into each PML-NB after removal of As^{3+} or Sb^{3+} from culture medium. Both “aggregation” and “agglomeration” are scientifically defined terms. Then, we hope that we do not need to explain the difference between these terms.

We have removed "occasionally found" and rewritten the corresponding sentence as follows. “Although most PML-NBs of HEKGFPPML cells were granular dot-like, a small percentage (0.5%) of HEKGFPPML cells accommodated unstable toroidal PML-NBs (Fig. 2B).”

Q6: page 9, last sentence before Fig. 3 legend: ..: a possibility that that...

A6: We have amended the duplication.

Q7: Fig 5A: the m of agglomerates is missing in the vertical legend of the graph

A7: We have corrected the spelling for agglomerates in Fig. 5A and uploaded the revised Fig. 5.

Q8: Fig 7: the authors must comment why there is less immunoprecipitated GFPPML from the soluble fraction of untreated cells than from that of As-treated cells. Shouldn't the opposite be expected according to the results shown in Fig.3?

A8: We think that this is one of the most important points in PML biology. Most GFPPML and SUMOylated GFPPML proteins shifted from the RIPA-soluble to the RIPA-insoluble form after 2 h-exposure to arsenic as demonstrated in Fig. 3. Thus, we cannot expect an enough amount of GFPPML proteins in the immunoprecipitate prepared from the soluble fraction of the As-exposed (2 h) cell lysate. As we show in Fig. 6, however, substantial

amounts of GFPPML and SUMOylated PML still remained in the RIPA-soluble fraction when the lysate was prepared from the cells after 1 h-exposure to As. If the immunoprecipitation sample is prepared from cells after 2 h-exposure to As^{3+}, the amount of GFPPML should be much less than that of untreated cells. That is why we prepared the immunoprecipitation samples after 1 h-exposure to As^{3+} to show that a substantial amount of SUMOylated PML remained in soluble fraction. Ubiquitinated PML was also present in the soluble fraction. We hope that we clearly described that the exposure time was 1 h for the collection of immunoprecipitation samples referring to Fig. 6.

As to non-SUMOylated GFPPML, a larger amount of GFPPML should have been immunoprecipitated from the lysate of untreated cells than from that of As^{3+}-exposed cells as the reviewer pointed out. For now, we do not have good experimental data to answer this question. However, it is plausible that non-SUMOylated GFPPML co-precipitated efficiently with a huge amount of SUMOylated GFPPML in the lysate of As^{3+}-exposed cells. Arrows indicating non-SUMOylated GFPPML have been moved a little downward in Fig. 7B, because they did not point non-SUMOylated GFPPML in the previous submission.

Q9: Fig 8: the meaning of the superscript symbols used in the tables should be explained
A9: We apologize for the missing part. We have added annotations for each superscript symbol as follows: *, Significantly different from control group. #, Significantly different from all other groups. &, Significantly different from control and TAK243 groups.

Q10: legend of S2 refers to the legend of S2
A10: We apologize again. We have revised S2. Fig.

Reviewer #2:

Q1-1: I had asked the authors to precisely define and quantify the different types of bodies they observe. In response to this request, the authors simplified the naming of the different structures. Still, I have difficulties with the qualitative distinction that is made between the toroidal *structures vs the "punctates"* (actually, although I am not a native English speaker, I am not sure that using the word "punctate" as a noun is appropriate: I think it is mainly used as an adjective). These two types of structures are very difficult to distinguish on the Figure images, which makes this distinction difficult to understand and evaluate.
A1-1: We understand that our manuscript had seriously suffered from ambiguous names of PML bodies since Reviewer 1 raised the same concern (Q5 of Reviewer 1). As far as

we know many names have been used to describe “phase-separated” PML such as nuclear bodies, dots, speckles, structures, punctates, and aggregates in the literature. Unfortunately, there is no clear definition for these terms. We have removed “structures” and “punctates” from the revised manuscript and simplified the terminology for PML bodies as far as reasonably possible. See also A5, an answer to Q5 of Reviewer 1.

Q1-2: Wouldn’t it be better (and more meaningful?) to categorize the different types of structures by their apparent size on 2D images (or volume when 3D information is available) rather than by their qualitative shape (toroidal vs punctate)? Furthermore, the authors could then use this metrics all along their manuscript to more quantitatively describe the transitions that they observe during the cell cycle and after heavy metal treatment. I believe this would be more rigorous and informative than the current classification.

A1-2: We wish that we could categorize different types of PML assemblies using quantitative indices. We once tried to estimate the volume of toroidal PML-NB using a formula for calculation of the torus volume (See the figure below). Moreover, quantitation of GFPPML in each PML-NB is technically possible if the GFP intensity of each pixel is integrated in the volume calculation.

	Small toroid inner (nm)	Small toroid outer (nm)	Large toroid inner (nm)	Large toroid outer (nm)
	302	605	660	1240
	138	522	552	1210
	261	646	523	1350
	179	646	440	1340
	297	591	443	1180
	220	596	688	1290
	206	522	577	1180
Ave	229.0	589.7	554.7	1255.7
SEM	23.1	19.4	36.5	27.2

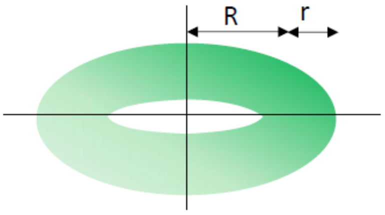

$$V=2\pi^2r^2R$$

Diameter of small toroid (nm)		Diameter of large toroid (nm)	
Inner	Outer	Inner	Outer
229±23.1	589±19.4	555±36.5	1256±27.2

However, the size and shape of PML-NBs and peri-nuclear PML aggregates vary dynamically, and they are not symmetrical. Moreover, some PML aggregates appear to be porous. Thus, we concluded that an average diameter and shape better describe the figure of PML assembly as we simply describe, for example, the nucleus using an average diameter and shapes.

Q2-1: Reviewer 1 asked the authors to “extensively rewrite” the manuscript and stressed

that “the reading is sometimes difficult”. Similarly, I asked the authors to “better explain the significance of their findings”. Although some text changes have been made, the overall manuscript remains difficult to read and the conveyed messages are not always clear. In my opinion, more efforts could and should be made to more clearly explain the context of the study, the rationale of the experiments, the main findings and their biological implications. Actually, in some cases the implemented text changes did not improve clarity. For instance, I do not understand the meaning of the new sentence “the SUMO-SIM interaction, which is implicated in liquid-liquid phase separation [32], occurs only when PML-VI is SUMOylated” (page 4) which the authors added in response to a question of reviewer 1. How can there be a “SUMO-SIM” interaction if PML-VI has no SIM ? Do the authors refer to interactions with the SIM of other proteins?
A2-1: The SUMO-SIM interaction is a mutual contact. Even though PML-VI does not have a SIM motif, SUMOylated PML-VI can associate via SUMO-SIM interaction with other proteins that have SIM motifs. This is an important point because the SUMO-SIM interaction on PML occurs only when PML-VI is SUMOylated. Many scientists studying biomolecular condensates believe that SUMO-SIM interaction leads to phase separation and form granules or dots like PML-NBs in cells. The present study clearly shows that PML-VI formed PML-NBs without clearly detectable SUMOylation on PML in untreated cells. We have added “between PML and the other proteins” after “the SUMO-SIM interaction” to avoid misunderstanding.

We have stressed on several important findings in our 2nd revised manuscript. See also A0 (an answer to Q0 of Reviewer 1).

Q2-2: As another example, the authors replaced in the introduction the term “life-time” by “residence-time” (page 2). Indeed, as noted by reviewer 1, the term “life-time” was not correct. But the authors omitted to rephrase the rest of the sentence, which in my opinion is still unclear. To clarify this sentence, the authors simply need to explicitly write that what they describe is the “residence-time of PML in nuclear bodies”. Thus, I really encourage the authors to make further efforts to improve the clarity of their manuscript.
A2-2: Reviewer 1 pointed out the same problem in our previous manuscript (Q1 of Reviewer 1). We have corrected the terms as suggested.
