# Supplementary material for: Effects of arsenic on the topology and solubility of promyelocytic leukemia (PML)-nuclear bodies
Source: PLoS One. 2022 May 20;17(5):e0268835. doi: 10.1371/journal.pone.0268835 (PMC9122205; doi:10.1371/journal.pone.0268835)
Supplement: Attachment — Submitted filename: Response to Reviewers.docx [file pone.0268835.s008.docx]

Dear Prof. Claude Prigent,

We would like to thank the Editor and reviewers for their valuable comments on our manuscript entitled “Effects of arsenic on the topology and solubility of promyelocytic leukemia (PML)-nuclear bodies”. We have addressed all reviews’ questions and the Journal Requirements inquiries, and have rewritten the manuscript according to their suggestions. We hope that our revised manuscript is now acceptable for publication in PLoS ONE.

Best regards,

Seishiro Hirano.

**Re: Journal Requirements**

Q1. Please ensure that your manuscript meets PLOS ONE's style requirement.

A1. We are sure that our revised manuscript fully meets PLOS ONE's style requirements.

Q2. Please remove any funding-related text from the manuscript and let us know how you would like to update your Funding Statement.

A2. We have removed the funding information from the Acknowledgments section. No further action is required because the same statement appears in the Financial Disclosure Column.

Q3. Upon re-submitting your revised manuscript, please upload your study’s minimal underlying data set as either Supporting Information files or to a stable, public repository and include the relevant URLs, DOIs, or accession numbers within your revised cover letter.

A3. Data Availability statement: The minimal data set is provided as Supporting Information files. Raw data used for preparation of all graphs are provided as an Excel Book file named “Raw data for graphs”.

Q4. PLOS ONE now requires that authors provide the original uncropped and unadjusted images underlying all blot or gel results reported in a submission’s figures or Supporting Information files.

A4. We have provided all original western blot full images as a Supporting Information named ‘S1_raw_images’. The images were converted to annotated Tiff files with LZW compression. Then, they were compiled as a single PDF.

Q5. Please review your reference list to ensure that it is complete and correct.

A5. The reference list has been updated for PLoS reference style in the revised manuscript.

**Point-to-point responses to Reviewers’ comments**

**Reviewer #1**

Q1: The authors extensively rewrite it to better highlight the biological questions they want to address and the original results they obtained, while reducing the length given to what is already known.

A1: In DISCUSSION (page 16), we have removed several sentences, and added the following sentences highlighting the As^3+^-induced agglomeration of PML-NBs since this is the first report on quantitative analysis of the agglomeration of PML-NBs, and the agglomeration appears to be closely related to the solubility change and SUMOylation of PML; “It should be noted that prolonged exposure to As^3+^ caused agglomeration of PML-NBs which is reversible after removal of As^3+^ from the culture medium (Figs. 4 and 5). To our best knowledge, this is the first quantitative study to report the agglomeration of PML-NBs. Since the agglomeration of PML-NBs was observed 4 h after exposure to As^3+^, either the solubility change or SUMOylation of PML or both may lead to the agglomeration.” Accordingly, CONLUSIONS has been rewritten stressing the As^3+^-induced agglomeration of PML-NBs.

In ABSTRACT, the last part was replaced with the following sentence: ” These results warrant the importance of As^3+^- or Sb^3+^-induced solubility shift of PML for the regulation intranuclear dynamics of PML-NBs.”

Q2: The authors analyze in the manuscript the isoform VI of PML, which is lacking a SUMO-interacting motif present in the other isoforms. Isn't this choice a concern when studying the solubility and the SUMOylation of the protein? I think this point should be discussed.

A2: The following sentence has been added to the last paragraph of INTRODUCTION. so that the readers easily understand the reason that we used this PML isoform in the present study. “PML-VI is the only nuclear PML isoform that lacks a SIM motif, and therefore the SUMO-SIM interaction, which is implicated in liquid-liquid phase separation [32], occurs only when PML-VI is SUMOylated.”

( 32.	Patra U, Muller S. A Tale of Usurpation and Subversion: SUMO-Dependent Integrity of Promyelocytic Leukemia Nuclear Bodies at the Crossroad of Infection and Immunity. Front Cell Dev Biol. 2021;9:696234. doi: 10.3389/fcell.2021.696234. PubMed PMID: 34513832; PubMed Central PMCID: PMC8430037.)

Q3: The quality and resolution of the microscopy images is low (as judged from the pdf available for reviewing) and should be increased. Actually, it would be useful for many readers to display the exact contour of the nuclei, either by outlining them in the images of by adding photos of a DAPI (or equivalent) staining.

A3: The quality of micrographic photos has increased so that readers can see the shape of both nuclei and PML-NBs clearly. We first tried to outline the nuclei to make the nuclear contours clear. However, the higher quality of the photos now displays the contour of the nuclei clearly in the bright field images. Thus, we did not modify the microscopic photos.

Q4: I am not sure of the pertinence of testing the possible inhibition of the proteasome by As3+ and Sb3+ on 20S proteasome only. Indeed, one could imagine that these compounds inhibit more the 26S than the 20S proteasome, by interfering not directly with the proteolytic activities but with other activities or properties of the 26S proteasome. To strengthen this point, one possibility would be to better document that there is no accumulation of ubiquitylated proteins in cells upon these treatments, as indicated by the input fractions in Fig.7.

A4: We understand this reviewer’s suggestion. However, the input bands shown in Fig. 7 are not quantitative data. Then, we have added the following adverbial clause; “although we do not deny a possibility that that As^3+^ and Sb^3+^ affect the 26S moiety of proteasomes and decrease the UPS activity.”

Q5: If overall the quality of the English is adequate, the reading is sometimes difficult, and it seems to me that the manuscript would benefit from editing by a native English speaker to make the style clearer. As it is, the meaning and therefore the conveyed message of some sentences is sometimes obscure. For example, maybe I missed the point, but I could not understand the sentence "suggesting that the topology was retained in the extranuclear region" in the first paragraph of the results. The use of the term 'life-time' in the introduction is not correct. The authors probably meant 'residence-time'? Fig 10 legend: SUMO2/3 is more (not less) abundant in peri-nuclear PML aggregates in ML792-treated cells

A5: Ambiguous phrases and sentences including typos have been corrected as suggested by the reviewer.

**Reviewer #2**

Q1: The authors have studied the topology of PML nuclear bodies and their dynamics in two cell lines. They have quantified the size and number of PML bodies, but most of their other observations on the shape of the bodies, remain qualitative, poorly documented and difficult to visualize in the figures that are provided. They should more precisely define the different types of bodies they mention (toroid bodies, annular bodies, amorphous bodies, small dots, …) and provide metrics to characterize these bodies and their dynamics.

A1: We apologize for the confusion. We used annular PML-NB and toroidal PML-NB for the same structure in the first submission. The words “annular PML-NBs”, “amorphous”, and “dots” have been removed from the revised manuscript. However, “punctate” is used when necessary in the revised manuscript.

Q2: The authors should complete their discussion and conclusion to better explain the significance of their findings on the understanding of PML biology.

Q2: Reviewer 1 also pointed out that the significance of our current study was not well-documented. We have revised the manuscript highlighting the agglomeration of PML-NBs following longer exposure to As^3+^. See A1 to Reviewer 1’s Q1 for our reply to this comment.

Q3: Figure 1 and Figure S1: The quantification provided in Figure S1 is important and should be included in the main Figure rather than in the supplementary data.

A3: The whole content of Figure S1 in the first submission is now a part of Fig.1 in the revised manuscript. Accordingly, the other Supplementary figures have been renumbered and the legend to Fig. 1 has been changed.

Q4: The PLOS data policy requires authors to make all data underlying the findings described in their manuscript fully available. Thus, the individual data points used for the plots of Figures 3A, 4A, 8A, 8B, S1 S4 and S6 should be provided, either as scatter plots or in supplementary tables.

A4: All raw data for Figs. 1 (S1 Fig. in the first submission),3A, 5A, 8A, 8B, S3 (S4 in the first submission), and S5 (S6 in the first submission) are shown as an Excel Book and uploaded as a Supplemental file named “Raw data for graphs”. Note that Supplementary Fig. 1 is now a part of Fig. 1 according to the reviewer’s suggestion. The reviewer probably intended to write 5A instead of 4A because there was no graph in Fig. 4.

Q5: The legend of Figure 8 should state whether the indicated variations of PML nuclear bodies number and size are SD or SEM. Actually, I encourage the authors to use SD rather than SEM as summary statistics for this and all other figures as this enables the reader to evaluate data dispersion.

A5: We understand that there is always a debate about which should be used to present data dispersion, SD or SEM. Some scientists prefer SEM to SD because SEM is always smaller than SD. SD measures the dispersion of a population. SEM measures the standard deviation of the set of means for the population mostly in Gaussian distributions. That is why we usually use SEM for in vitro data when the sample size is small. Thus, data are presented as means ± SEM in the present study. We provided both SD and SEM values in the Supplementary data (Excel, Raw data for graphs) for Fig. 1 to avoid the long-standing debate.

Q6: Figure annotations: The annotations of many figures could be improved to facilitate their understanding and interpretation. For instance, in Figure 1 the panel A and B represent similar experiments performed with two different cell lines (CHOGFPPML and EKGFPPML). The authors should include the names of these cell lines in the corresponding panels of this (and other) Figure(s). Similarly, in Figure 5B, the lanes 1,2,3 and 4 correspond to different time points of As^3+^ treatment. This could be explicitly notated in the Figure panel rather in the legend. These two examples are not exhaustive, and I encourage the authors to think for each Figure panel whether more information could be provided to improve their legibility.

A6: We have added some annotations in Figs. 1, 2, 5B, 6, 8, 9, and S4 (S5 in the first submission) so that readers easily and rapidly understand figures.

Q7: The authors should state the mechanism of action of TAK243 and ML792 in the main text.

Q7: We have added a short description about these mechanism-based inhibitors in DISCUSSION as follows; TAK243 (an inhibitor of UBA1-Ub thioester bond formation) and ML792 (an inhibitor of UBA2-SUMO thioester bond formation)
